# Sitting Time and Body Mass Index in Diabetics and Pre-Diabetics Willing to Participate in a Lifestyle Intervention

**DOI:** 10.3390/ijerph8093747

**Published:** 2011-09-19

**Authors:** Judith H. M. Helmink, Stef P. J. Kremers, Femke N. van Brussel-Visser, Nanne K. de Vries

**Affiliations:** 1 Department of Health Promotion, Maastricht University, School for Nutrition, Toxicology and Metabolism (NUTRIM), 6211 LK, The Netherlands; E-Mail: judith.helmink@maastrichtuniversity.nl; 2 Netherlands Institute for Sport and Physical Activity, Ede 6717 LZ, The Netherlands; E-Mail: Femke.vanbrussel@nisb.nl; 3 Department of Health Promotion, Maastricht University, School for Public Health and Primary Care (CAPHRI) and School for Nutrition, Toxicology and Metabolism (NUTRIM), 6211 LK, The Netherlands; E-Mail: n.devries@maastrichtuniversity.nl

**Keywords:** sitting time, physical activity, type 2 diabetes, lifestyle intervention, motivation to be physically active, Body Mass Index

## Abstract

This cross-sectional study examined the relationship between Body Mass Index (BMI), total sitting time and total physical activity time in a generally overweight or obese population of type 2 diabetics or pre-diabetics willing to participate in a lifestyle intervention [n = 221, 55.1% male, mean age (SD) 62.0 (9.9), mean BMI (SD) 31.4 (5.0)]. In addition, we aimed to identify demographic and psychosocial associates of the motivation to become more physically active. The measurement instrument was a self-report questionnaire. Results showed that total sitting time was more closely related to BMI than total physical activity time. Subjects with a higher weight status were more sedentary, but they were also more motivated to be physically active. On the other hand, their self-efficacy to be physically active was lower than subjects with a lower weight status. Lifestyle interventions to decrease the risk of obesity and type 2 diabetes should aim not only at increasing total physical activity time, but also at reducing the total sitting time. Despite generally high levels of motivation among these obese participants, intervention designers and intermediaries should be aware of their low level of self-efficacy towards being physically active.

## 1. Introduction

Obesity and physical inactivity are the most important risk factors for developing type 2 diabetes mellitus. In the Netherlands, five million people are considered overweight and/or physically inactive [1]. A total of 740,000 people were diagnosed with diabetes in 2007 in the Netherlands, 90% of them with type 2 diabetes [2]. The incidence of type 2 diabetes is increasing worldwide, and the quality of life of people with diabetes is lower than that of people without this chronic disease, especially as regards well-being and physical functioning [3]. Hence, measures to prevent type 2 diabetes have been advocated [4], with lifestyle interventions focusing on the promotion of a healthy diet and physical activity (PA) [5–11].

Lifestyle interventions should not only aim to increase PA, but also to decrease sedentary behaviour, as moderate to vigorous activity a few times a week will not compensate for the potential negative effects of excessive sedentary behaviour [12]. Sedentary behaviour refers to activities that do not increase energy expenditure substantially above the resting level, such as sitting, lying down or viewing TV [13]. It has been suggested that sedentary behaviour should be explicitly measured instead of being defined as a lack of PA [13,14]. In fact, defining a sedentary population based on low levels or absence of PA might be inaccurate, as sedentary behaviour and PA are two independent behaviours with different effects on health outcomes [15,16]. A review by Proper and colleagues showed that there was moderate evidence for an independent relationship between sedentary behaviour and type 2 diabetes [17].

Non-exercise active thermogenesis (NEAT) can play a key role in the energy expenditure of obese people. NEAT is the energy expenditure associated with everyday activity [18]. Altering one’s postural allocation from a seated to a standing position or engaging in light ambulation has been shown to significantly increase energy expenditure. A difference in NEAT has been observed between obese and lean subjects [18]. Obese subjects have been shown to sit for 2.5 hours/day more than lean subjects [18,19].

Underlining the importance of sedentary behaviour in explaining weight status, Santos and colleagues [20] demonstrated that in a general population of Portuguese men, even those meeting public health guidelines for leisure time PA may be at risk for overweight and obesity if they spend a large proportion of their leisure time in sedentary activities. They found that men with a high total sitting time and with sufficient PA time had a higher average Body Mass Index (BMI) than those with low total sitting time and sufficient PA time. The difference between the groups was statistically significant, though with a small effect size (Cohen’s d < 0.1) [20]. In line with the empirical evidence from research about NEAT, we hypothesised that an even larger share of the BMI would be explained by low sitting time in a subpopulation of generally overweight or obese diabetics or pre-diabetics.

The results of the study by Santos *et al.* [20] also indicated that total sitting time was positively associated with BMI after adjustment for some potential confounders (including total PA time), but they did not correct for potentially relevant cognitive determinants of PA. Theories about behavioural determinants generally distinguish three types: outcome expectancies or attitude, subjective norm and self-efficacy. Attitudes are determined by the beliefs about attributes of performing the behaviour [21]. Outcome expectancies are a person’s estimate that a given behaviour will lead to certain outcomes [22]. Subjective norm reflects expectations about what people in our social environment want us to do [23]. Self-efficacy (also known as perceived behavioural control) refers to a person’s expectation that he or she has the ability to implement the desired behaviour [24].These cognitions have been successfully applied in research on PA [25–29].

The aim of the present study to examine the relationship between BMI, total sitting time and total PA time in a generally overweight or obese population of diabetics or pre-diabetics, while controlling for demographic and psychosocial associates of the motivation to become more physically active by participating in a lifestyle intervention.

## 2. Methods

### 2.1. Study Design and Sampling

Participants in this study were people who had agreed to be included in a lifestyle intervention programme called BeweegKuur (see [11] for more information). People with an impaired fasting glucose (pre-diabetics; *i.e.*, fasting glucose range (finger prick) ≥ 5.6 mmol/L–< 6.9 mmol/L) and persons with type 2 diabetes (HbA1c ≥ 7.0) were eligible for inclusion in the intervention. Exclusion criteria were type 2 diabetes with three or more complications, type 2 diabetes with serious poly-pharmacy and type 2 diabetes with type 3 hypertension. In this programme, a patient’s general practitioner (GP) determines whether the intervention will be offered. After the patient agrees to participate, coaching and supervision is provided by a lifestyle advisor (LSA, often the GP’s assistant), based on principles of Motivational Interviewing [30].

The present study used a cross-sectional non-controlled design, based on the (self-reported) baseline data of a longitudinal monitoring study. The LSA was responsible for the distribution of the questionnaire to the patients, who received it at the end of their intake consultation with the LSA. Patients also completed an informed consent form. The questionnaire could be returned to the university free of postage, and participants could win a gift voucher. The questionnaire was completed by 361 persons.

### 2.2. Measures

Demographic variables assessed in the questionnaire were date of birth, educational level, gender and country of birth. Self-reported weight and height were used to calculate BMI [weight (kg)/height (m)^2^].

PA and sitting time were measured with the short version of the International Physical Activity Questionnaire (IPAQ) [31]. Based on the ACSM/AHA physical activity guidelines, subjects were categorised as: insufficiently active (participants who reported fewer than 150 min/week of at least moderate-intensity PA or less than 20 min/week of vigorous-intensity PA) or sufficiently active (participants who reported 150 min/week or more of at least moderate intensity PA or 20 min/week or more of vigorous-intensity PA) [32]. Total PA time in this article was measured by calculating the amount of moderate and vigorous PA time. The proxy measure of sedentary behaviour was the time spent sitting on an ordinary weekday. This was measured with the question: ‘During the last 7 days, how much time did you spend sitting on a week day?’ where the respondents could fill out the hours or minutes spent sitting. The sitting time includes time spent at work, at home, while doing course work and during leisure time. Outliers (sitting time on an ordinary day < 120 min/day) were recoded to the 5th percentile. Subjects were categorised as having low or high total sitting time, based on the median value for total sitting time found in this sample (360 min/day). Participants were also categorised into the following four groups: low total sitting time/sufficient PA time; low total sitting time/insufficient PA time; high total sitting time/sufficient PA time and high total sitting time/insufficient PA time. Subjects with missing data on any of the PA measures (n = 140; 38.8%) were removed from the further analyses. Drop-out analyses showed that the persons who did not fill in the PA measures did not differ from the complete cases, except for education (more highly educated people being more likely to fill in the PA measures).

The questionnaire items regarding the psychosocial determinants of PA in the BeweegKuur programme were based on the Theory of Planned Behaviour [23], Social Cognitive Theory [33] and the ASE model [24]. Outcome expectancies were measured with eleven items, with answers on a 5-point scale (‘I do not agree at all’ (1) to ‘I fully agree’ (5)). Reliability analysis yielded a Cronbach’s alpha of 0.79. An example of an item was ‘If I participate in BeweegKuur, I will lose weight’. Six items measured the attitude concept (α = 0.65). An example of a question was: ‘The BeweegKuur programme suits people like me’. Five self-efficacy questions (α = 0.87) were asked, again using a 5-point scale with answer categories ranging from ‘I do not agree at all’ (1) to ‘I fully agree’ (5). An example of a question was ‘I think I will be able to attend the whole BeweegKuur programme.’ Three questions were asked regarding subjective norm, measured on a 5-point scale (‘I do not agree at all’ (1) to ‘I fully agree’ (5) (α = 0.87). An example of these questions was ‘My family wants me to participate in BeweegKuur’. Motivation to become more physically active at the start of the BeweegKuur intervention was assessed with the question ‘How motivated are you to be more physically active’, with answering categories on a scale from 0 (not motivated) to 10 (extremely motivated).

### 2.3. Statistical Analyses

The statistical analyses were conducted using SPSS 17.0. Frequencies were determined to examine the characteristics of the sample and the distributions of age, gender, education, occupation, BMI, sitting time, total PA time, outcome expectancies, attitude, self-efficacy and subjective norm in the sample. Cognitive, motivational and behavioural responses were explored using a Chi-square test and correlations. We correlated demographic characteristics with intermediate (cognitive factors) and ultimate outcomes (total sitting time, total PA time, obesity and BMI). Linear regressions were conducted with BMI as the dependent variable, while independent variables were demographic characteristics (gender, age, education, occupation, country of birth) as well as cognitive factors, total sitting time and total PA time. Logistic regressions were conducted with weight status (obese (1) *vs.* non-obese (0)) as the dependent variable, and with the same independent variables as the linear regression with BMI. Demographic and psychosocial associates of the motivation to become more physically active were assessed using linear regressions, with motivation as the dependent variable and demographic characteristics, cognitive factors, total sitting time, total PA time and obesity as independent variables. P-values below 0.05 were considered statistical significant, p-values below 0.10 were considered being a trend.

## 3. Results

More than half of the respondents were men (55.1%), with a mean age of 62.0 years. The average BMI was 31.4 kg/m^2^, and 60.6% of the respondents were obese. The average self-reported PA time was 0.53 hours/day and the average self-reported total sitting time was 6.41 hours/day. The motivation to be physically active was 7.66 (see Table 1).

As regards the sitting time/PA time subgroups, the subjects with low sitting time and sufficient PA had the lowest BMI, while the respondents with high sitting time and sufficient PA time had the highest BMI. The difference between these groups was statistically significant (p = 0.03; Cohen’s *d* = 0.42) (Table 2).

Correlation analyses showed that more highly educated people and those who were employed had a higher sitting time, while homemakers had a lower sitting time. Respondents with higher sitting times were less physically active. Higher BMI levels were more prevalent among the younger age group, low-educated respondents, those in employment, respondents from non-Western countries and those with a high sitting time (but not a high PA time). Weight status was also positively correlated with outcome expectancies and subjective norm (Table 3).

Linear regression analyses showed that a higher BMI was negatively associated with age and education. Younger participants had a higher BMI than older participants. Respondents with a medium-level or high education had a lower BMI than those with a lower education.

Logistic regression showed that the BMI groups were associated with age, self-efficacy and sitting time. Younger respondents were more likely to be in the obese group than older ones. Respondents in the lower BMI group had higher self-efficacy and spent less time sitting than obese subjects.

Linear regressions with the motivation to be physically active showed that people who had a medium-level education were more motivated than the lower educated respondents. A higher motivation was associated with higher self-efficacy. Obese respondents were more motivated to be physically active than those in the lower BMI group (see Table 4).

## 4. Discussion

The results for this specific group of diabetic or pre-diabetic patients with overweight or obesity showed that the total sitting time was more closely related to BMI than total PA time. Subjects with high sitting time had a higher BMI, even after correction for total PA time. This is in line with the results of Santos and colleagues [20] who demonstrated that, after adjusting for potential confounders (including total PA time) total sitting time was positively associated with BMI. In fact, the differences between the groups in our study were even larger than the differences demonstrated by Santos and colleagues. Furthermore, our results showed that subjects with high total sitting time and sufficient total PA time had the highest BMI, and that, after adjustment for potential confounders (including total PA time and cognitive determinants of being physically active), sitting time was positively associated with being obese. We therefore conclude that even those diabetics or pre-diabetics who meet public health guidelines for leisure time PA may be at risk for co-morbidities if they spend a large proportion of their residual leisure time in sedentary activities. These results indicate that it is important in future lifestyle interventions to aim not only at increasing PA but also at decreasing total sitting time. Because of the key role NEAT plays with obese people, it will be beneficial if sedentary participants of lifestyle interventions will succeed in decreasing their sedentary behaviour.

Sedentary behaviour has been shown to be a risk factor for type 2 diabetes and increased mortality [34]. Various studies have investigated the effects of television viewing on BMI in type 2 diabetes patients [34–37]. In line with our results, Dunstan and colleagues showed that even when people met the exercise guidelines, television viewing was associated with weight gain [36]. Another study showed that women who spent a considerable amount of time watching television had an increased risk of type 2 diabetes at every level of PA [37]. A similar relationship was found in a study with men [35], where men who were obese and physically active had a substantially increased risk for type 2 diabetes compared with those who were lean and inactive, while obese and inactive men were at highest risk [35].

Although our selection method meant that all subjects in our sample were motivated to be physically active, our results indicated that subjects with a higher BMI were more motivated and demonstrated a more positive attitude towards participating in a lifestyle intervention than the subjects with a lower BMI. However, obese subjects also had lower self-efficacy regarding successful participation in lifestyle interventions compared to those with a lower BMI. This is in line with the results discussed in a review by Allen [26], who reported that different exercise studies with diabetes patients had found a significant relationship between self-efficacy and exercise behaviour [26]. A study by Plotnikoff and colleagues [29] also reported that self-efficacy was more important than outcome expectancies in predicting PA; their results supported the need to target self-efficacy to set goals and change behaviour in PA promotion [29]. Participation in PA programmes has been shown to improve self-efficacy and reduce mental health problems, which can have effects on the quality of life [38].

Various strategies can be used to increase self-efficacy regarding PA, such as goal-setting, providing training and guidance in performing activities, demonstrating the desired behaviour, giving verbal reinforcement and reducing anxiety [39]. Giving feedback by telephone or clinical follow-up is also a strategy to increase self-efficacy [40]. These strategies are all part of the BeweegKuur intervention. Participants in this intervention are guided by an LSA, who has been trained in Motivational Interviewing, based on the principles formulated by Milner and Rollnick [30]. This technique is expected to enable LSAs to raise the patients’ confidence about increasing their PA and decreasing their sedentary behaviour. Goal-setting is part of the counselling process. Participants receive a logbook, in which they can enter their goals and record the time spent on PA. These goals and reports are then discussed with the LSA. Furthermore, participants can start the intervention in an exercise setting with a physiotherapist, enabling the latter to show the participants that it is safe to be physically active [11].

One limitation of the present study was its cross-sectional, non-controlled design. This implies that we cannot draw any conclusions on the causality of the identified associations. Another limitation was the use of self-report measures of weight, height, PA and sitting time, which can lead to underestimation (weight) and overestimation (PA time) as well as socially desirable answers. In addition, we were unable to make distinctions between sitting time in different settings (e.g., at work or during leisure time) which are likely to vary in terms of control over behaviour change. The best way to avoid this in future studies would be to combine self-reported measurements with objective measurements. A further limitation was that all participants in this study had voluntarily subscribed to participate in a lifestyle intervention, implying high levels of motivation among the participants. Since they participated in a lifestyle programme, it is possible that the obese subjects were aware of their high weight status and wanted to lose weight by participating in the intervention. Longitudinal measurements are needed to show whether the higher level of motivation results in larger intervention effects in the obese subjects. Finally, the small sample size was a limitation. Therefore, separate analyses for gender and age groups could not be conducted. Strengths of the study include the relatively large sample of diabetic or pre-diabetic patients and the use of social-cognitive factors in explaining weight status.

## 5. Conclusions

In conclusion, our results from cross-sectional analyses showed that it was not total PA time but total sitting time which was best associated with BMI among diabetics or pre-diabetics. Obese diabetics or pre-diabetics had higher sitting time, but were also more motivated and had a more positive attitude towards participating in a lifestyle intervention, compared to non-obese participants. But these subjects also had a lower self-efficacy regarding successful participation in a lifestyle intervention. Lifestyle interventions should not only aim to increase total PA time, but also to reduce the total sitting time, to decrease the risk of obesity and type 2 diabetes.

## Figures and Tables

**Table 1:** Descriptive characteristics of the participants (n = 221).

	%	Mean ± SD		%
**Gender**			**Level of education**	
Male	55.1		Low education	47.3
Female	44.9		Medium-level education	30.5
**Age**		62.0 ± 9.9	High education	22.3
			**Occupation**	
**BMI (kg/m^2^)**		31.4 ± 5.0	Employed	34.2
BMI > 30 kg/m^2^	60.6		Homemaker	21.0
**Total PA time (hours/day)**		0.53 ± 0.87	Retired	33.3
**Total sitting time (hours/day)**		6.41 ± 2.93	Unemployed/disabled	11.4
**Outcome expectancies** (1–5)		3.91 ± 0.40		
**Attitude** (1–5)		3.90 ± 0.41		
**Self-efficacy** (1–5)		3.96 ± 0.53		
**Subjective Norm** (1–5)		3.38 ± 0.82		
**Motivation to be physically active** (1–10)		7.66 ± 1.43		

**Table 2:** Mean (±SD) BMI (kg/m^2^) by categories combining total sitting time and total PA time.

	BMI (N = 221)	BMI > 30 (%)
Low total sitting time/sufficient PA time (N =5 4)	30.3 ± 4.0 *	57.4
Low total sitting time/insufficient PA time (N = 42)	31.1 ± 4.7	57.1
High total sitting time/sufficient PA time (N = 58)	32.4 ± 5.9	65.5
High total sitting time/insufficient PA time (N=67)	31.7 ± 5.0	61.2

* p < 0.05 different from high total sitting time/sufficient PA time; PA = physical activity.

**Table 3:** Correlates of the demographic variables, cognitive factors, behavioural factors and weight status.

	Demographic characteristics										Cognitive factors				Behavioural factors		Weight status

	Gender^a^	Age	Education			Occupation				Country of birth b	Outcome expectancies	Attitude	Self-efficacy	Subjective norm	Total sitting time	Total PA time	Obesity
			Low	Medium	High	Employed	Homemaker	Retired	Unemployed								
Outcome expectancies	0.06	−0.20 **	−0.06	0.04	0.02	0.21 **	0.01	−0.04	−0.04	0.04							
Attitude	0.06	0.01	0.12	−0.09	−0.05	0.01	0.02	−0.13	−0.13	0.00	0.31**						
Self-efficacy	−0.01	−0.03	−0.05	0.07	−0.02	0.01	0.08	−0.10	−0.10	−0.01	0.51 **	0.36 **					
Subjective norm	0.00	−0.06	0.02	−0.07	0.06	0.11	0.02	−0.08	−0.07	0.12	0.23 **	0.25 **	0.21**				
Total sitting time	−0.17*	−0.09	−0.09	−0.08	0.19 **	0.15 *	−0.18 **	−0.00	−0.00	−0.04	−0.05	−0.03	−0.04	0.03			
Total PA time	−0.09	−0.05	0.03	−0.06	0.03	0.12	−0.08	0.06	0.06	−0.09	0.05	0.04	−0.07	0.02	−0.17*		
Obesity	0.11	−0.21 **	0.15 *	−0.11	−0.06	0.13	0.07	0.01	0.02	0.10	0.16 *	0.15 *	−0.01	0.13	0.10	0.01	
BMI	0.09	−0.33 **	0.18 **	−0.08	−0.14 *	0.14*	0.03	0.05	0.07	0.13 *	0.16 *	0.08	0.01	0.13 *	0.13 *	−0.04	0.74**

a Male (1); female (2),

b Netherlands (0); other country(1).

c BMI < 30 (0); BMI > 30 (1).

* correlation is significant at the 0.05 level.

** correlation is significant at the 0.01 level.

**Table 4:** Linear regression analyses with BMI and motivation to be physically active as dependent variable, and logistic regression with obesity as dependent variable.

	BMI kg/m^2^	Obesity (0/1)		Motivation to be physically active
	
	β	OR	95% CI	β
**Block 1**				
Gender a	0.07	1.48	0.64–3.40	−0.10
Age	−0.39***	0.95**	**0.90**–**1.00**	0.02
Education b				
*medium*	−0.15**	0.52	0.25–1.09	0.16**
*High*	−0.21**	0.48	0.20–1.15	−0.01
Occupation c				
*Homemaker*	0.04	1.13	0.34–3.70	0.01
*Retired*	0.10	0.95	0.34–2.68	−0.11
*Unemployed/disabled*	−0.00	0.47	0.15–1.51	−0.10
Country of birth d	0.04	1.84	0.40–8.47	−0.03
**Block 2**				
Outcome expectancies	0.09	1.88	0.70–5.05	0.15
Attitude	0.01	2.19	0.86–5.53	−0.03
Self-efficacy	−0.08	0.44**	**0.21**–**0.96**	0.40***
Subjective norm	0.08	1.17	0.76–1.81	−0.09
**Block 3**				
Total sitting time (hours/day)	0.12*	1.14**	**1.00**–**1.29**	0.01
**Block 4**				
Total PA time (hours/day)	−0.03	1.39	0.83–2.31	−0.05
**Block 5**				
Obesity e				0.13**
**R^2^**	0.21	0.20		0.31

a Male (1); female (2)

b reference category = low education

c reference category = employed

d Netherlands (0); other country(1)

e BMI < 30 (0); BMI > 30 (1).

*** < 0.01

** < 0.05

* < 0.10.

## References

[1] Poortvliet MC, Schrijvers CTM, Baan CA (2007). Omvang, Risicofactoren en Gevolgen, nu en in de Toekomst. Diabetes in Nederland.

[2] Baan CA, Schoemaker CG, Jacobs-van der Bruggen MAM, Hamberg-van Reenen HH, Verkleij H, Heus S, Melse JM (2009). Diabetes Tot 2025. Preventie en Zorg in Samenhang.

[3] Rubin RR, Peyrot M (1999). Quality of life and diabetes. Diabetes Metab Res Rev.

[4] Tuomiletho J, Lindström J, Eriksson JG, Valle TT, Hämäläinen H, Ilanne-Parikka P, Keinänen-Kiukaanniemi S, Laakso M, Louheranta A, Rastas M (2001). Prevention of type 2 diabetes mellitus by changes in lifestyle among subjects with impaired glucose tolerance. N Engl J Med.

[5] Laatikainen T, Dunbar JA, Chapman A, Kilkkinen A, Vartiainen E, Heistaro S, Philpot B, Absetz P, Bunker S, O’Neil A (2007). Prevention of type 2 diabetes by lifestyle intervention in an australian primary health care setting: Greater green triangle (ggt) diabetes prevention project. BMC Public Health.

[6] Mensink M, Feskens EJM, Saris WHM, de Bruin TWA, Blaak EE (2003). Study on lifestyle intervention and impaired glucose tolerance maastricht (slim): Preliminary results after one year. Int J Obes.

[7] Uutela A, Absetz P, Nissinen A, Valve R, Talja M, Fogelholm M (2004). Health psychological theory in promoting population health in päijät-häme, finland: First steps toward a type 2 diabetes prevention study. J Health Psychol.

[8] Absetz P, Oldenburg P, Hankonen N, Valve R, Heinonen H, Nissinen A, Fogelholm M, Talja M, Uuteka A (2009). Type 2 diabetes prevention in the ‘real world’: Three-year results of the goal implementation trial. Diabetes Care.

[9] Long BJ, Calfas KJ, Wooten W, Sallis JF, Patrick K, Goldstein M, Marcus BH, Schwenk TL, Chenoweth J, Carter R (1996). A multisite field test of the acceptability of physical activity counseling in primary care: Project pace. Am J Prev Med.

[10] Hosper K, Deutekom M, Stronks K (2008). The effectiveness of ‘exercise on prescription’ in stimulating physical activity among women in ethnic minority groups in the netherlands: Protocol for a randomized controlled trial. BMC Public Health.

[11] Helmink JHM, Meis JJM, de Weerdt I, Visser FN, de Vries NK, Kremers SPJ (2010). Development and implementation of a lifestyle intervention to promote physical activity and healthy diet in the dutch general practice setting: The beweegkuur programme. Int J Behav Nutr Phys Act.

[12] Hamilton MT, Hamilton DG, Zderic TW (2007). Role of low energy expenditure and sitting in obesity, metabolic syndrome, type 2 diabetes, and cardiovascular disease. Diabetes.

[13] Pate RR, O’Neill JR, Lobelo F (2008). The evolving definition of ‘sedentary’. Exerc Sport Sci Rev.

[14] Rosenberg DE, Bull FC, Marshall AL, Sallis JF, Bauman AE (2008). Assessment of sedentary behavior with the international physical activity questionnaire. J Phys Act Health.

[15] Biddle SJH, Gorely T, Marshall SJ, Murdey I, Cameron N (2004). Physical activity and sedentary behaviours in youth: Issues and controversies. J R Soc Promot Health.

[16] Sugiyama T, Healy GN, Dunstan DW, Salmon J, Owen N (2008). Joint associations of multiple leisure-time sedentary behaviours and physical activity with obesity in australian adults. Int J Behav Nutr Phys Act.

[17] Proper KI, Singh AS, van Mechelen W, Chinapaw MJM (2011). Sedentary behaviors and health outcomes among adults: A systematic review of prospective studies. Am J Prev Med.

[18] Levine JA (2007). Nonexercise activity thermogenesis—liberating the life-force. J Intern Med.

[19] Levine JA, Lanningham-Forster LM, McCrady SK, Krizan AC, Olson LR, Kane PH, Jensen MD, Clark MM (2005). Interindividual variation in posture allocation: Possible role in human obesity. Science.

[20] Santos R, Soares-Miranda L, Vale S, Moreira C, Marques AI, Mota J (2010). Sitting time and body mass index, in a Portuguese sample of men: Results from the Azorean Physical Activity and Health Study (APAHS). Int J Environ Res Public Health.

[21] Fishbein M, Ajzen I (1975). Belief, Attitude, Intention, and Behavior: An Introduction to Theory and Research.

[22] Bandura A (1977). Self-efficacy: Toward a unifying theory of behavioral change. Psychol Rev.

[23] Ajzen I (1991). The Theory of Planned Behaviour. Organ Behav Hum Decis Process.

[24] De Vries H, Dijkstra M, Kuhlman P (1988). Self-efficacy: The third factor besides attitude and subjective norm as a predictor of behavioral intentions. Health Edu Res.

[25] Salmon J, Owen N, Crawford D, Bauman A, Sallis JF (2003). Physical activity and sedentary behavior: A population-based study of barriers, enjoyment, and preference. Health Psychol.

[26] Allen NA (2004). Social cognitive theory in diabetes exercise research: An integrative literature review. Diabetes Edu.

[27] Delahanty LM, Conroy MB, Nathan DM (2006). Psychological predictors of physical activity in the diabetes prevention program. J Am Diet Assoc.

[28] Serrano E, Leiferman J, Dauber S (2007). Self-efficacy and health behaviors toward the prevention of diabetes among high risk individuals living in appalachia. J Community Health.

[29] Plotnikoff RC, Lippke S, Courneya KS, Birkett N, Sigal RJ (2008). Physical activity and social cognitive theory: A test in a population sample of adults with type 1 or type 2 diabetes. Appl Psychol.

[30] Miller WR, Rollnick S (1991). Motivational Interviewing: Preparing People to Change Addictive Behaviour.

[31] Craig CL, Marshall AL, Sjostrom M, Bauman AE, Booth ML, Ainsworth BE, Pratt M, Ekelund U, Yngve A, Sallis JF (2003). International physical activity questionnaire: 12-country reliability and validity. Med Sci Sports Exerc.

[32] Haskell WL, Lee IM, Pate RR, Powell KE, Blair SN, Franklin BA, Macera CA, Heath GW, Thompson PD, Bauman A (2007). Physical activity and public health. Updated recommendation for adults from the american college of sports medicine and the american heart association. Circulation.

[33] Bandura A (1986). Social Foundations of Thoughts and Action: A Social Cognitive Theory.

[34] Patel AV, Bernstein L, Deka A, Feigelson HS, Campbell PT, Gapstur SM, Colditz GA, Thun MJ (2010). Leisure time spent sitting in relation to total mortality in a prospective cohort of us adults. Am J Epidemiol.

[35] Hu FB, Leitzmann MF, Stampfer MJ, Colditz GA, Willett WC, Rimm EB (2001). Physical activity and television watching in relation to risk for type 2 diabetes mellitus in men. Arch Intern Med.

[36] Dunstan DW, Barr ELM, Healy GN, Salmon J, Shaw JE, Balkau B, Magliano DJ, Cameron AJ, Zimmet PZ, Owen N (2010). Television viewing time and mortality: The australian diabetes, obesity and lifestyle study (ausdiab). Circulation.

[37] Krishnan S, Rosenberg L, Palmer JR (2008). Physical activity and television watching in relation to risk of type 2 diabetes: The black women’s health study. Am J Epidemiol.

[38] Paxton RJ, Motl RW, Aylward A, Nigg CR (2010). Physical activity and quality of life—The complementary influence of self-efficacy for physical activity and mental health difficulties. Int J Behav Med.

[39] Bandura A (2004). Health promotion by social cognitive means. Health Edu Behav.

[40] Di Loreto C, Fanelli C, Lucidi P, Murdolo G, de Cicco A, Parlanti N, Santeusanio F, Brunetti P, de Feo P (2003). Validation of a counseling strategy to promote the adoption and the maintenance of physical activity by type 2 diabetic subjects. Diabetes Care.

